# Using Social Media Listening to Understand the Pressure Injury Experience: A Qualitative Descriptive Study

**DOI:** 10.2196/76682

**Published:** 2026-06-16

**Authors:** Jessica Zihui Song, Isabel Tsang, Parthena Mouratidis, Manahil Saeed, Alma A Ornelas L, Alaul Islam, Sharon Gabison

**Affiliations:** 1 KITE Research Institute, Toronto Rehabilitation Institute, University Health Network, Toronto, ON, Canada; 2 Rehabilitation Sciences Institute, Temerty Faculty of Medicine, University of Toronto, 500 University Avenue, Toronto, ON, Canada, 416-597-3422 ext. 7712; 3 Faculty of Liberal Arts and Science, Humber Polytechnic, Toronto, ON, Canada; 4 Department of Physical Therapy, Temerty Faculty of Medicine, University of Toronto, Toronto, ON, Canada

**Keywords:** social media listening, social media, pressure injury, pressure ulcer, bedsore, caregiver

## Abstract

**Background:**

Pressure injuries (PIs) are a common complication in people with reduced mobility or sensation and can be burdensome for individuals with PIs and their caregivers. Valuable insights and real-world challenges faced by individuals living with PIs can be captured through candid accounts posted on social media. Social media listening (SML) is a tool that can enhance the understanding of those with lived experience by offering firsthand accounts that are irreproducible from controlled studies.

**Objective:**

This study aims to capture the candid experiences of individuals with PIs and caregivers through social media.

**Methods:**

A noninterventional qualitative descriptive analysis was conducted using SML. Social media posts made on X (formerly Twitter), Reddit, and YouTube between January and December 2022 were compiled using SML tools X Pro (formerly TweetDeck) and Awario, and using Boolean search terms. Posts were manually screened for relevance, and duplicates were removed. Relevant posts were hand-coded by two independent reviewers. Inductive content analysis was used to analyze the posts.

**Results:**

The search yielded 666 relevant posts from 498 unique social media users. Most posts were made in the United States (170/666, 25.5%), the United Kingdom (150/666, 22.5%), and Canada (62/666, 9.3%). Social media users provided detailed descriptions of the PIs, including the setting in which the PI occurred, the cause of the PI, and how the PI was managed. The majority of PIs (197/666, 29.6%) were reported to have occurred in the hospital setting due to a perceived lack of care from care providers, and local wound care was often cited (99/666, 14.9%) as a PI management strategy. Three key themes were developed regarding living with or caring for someone with a PI: (1) challenges experienced when living with or caring for a PI, (2) needs related to PI prevention and management, and (3) emotions experienced when living with or caring for a PI. Social media users frequently discussed challenges associated with living with a PI, including negative personal impacts and poor perceived treatment quality. Users also described a critical need for health care, education, and social support. Finally, users often expressed anger and/or sadness related to living with or caring for a PI.

**Conclusions:**

SML captured candid insights into the experiences, challenges, and needs of individuals living with PIs and their caregivers globally that may not be gleaned from controlled studies. Individuals with lived experience and their caregivers often struggled with negative personal impacts regarding their physical health and daily functioning related to PIs, further highlighting the urgent need to address barriers to appropriate PI care. Clinicians and policymakers should consider practices and policies that optimize the delivery of person-centered PI care in order to overcome challenges and needs identified in this study.

## Introduction

Pressure injuries (PIs) occur due to damage to the integrity of the skin and underlying soft tissue resulting from severe and persistent localized pressure and shear, usually over a bony prominence [1-3]. Those at risk of developing PIs include older individuals and individuals with reduced mobility or sensation [1,3]. The global prevalence rate of PIs has remained stagnant at around 12% over the past few decades [4,5], which is notable because most PIs are avoidable if appropriate prevention techniques are applied [6]. This prevalence is especially troubling since PIs impose severe physical, mental, social, and emotional impacts on those living with or caring for them [7-11]. Individuals living with PIs often experience physical discomfort, social isolation, and challenges when performing daily activities [8]. Similarly, caregivers of individuals with PIs may experience a high caregiving burden and a low quality of life [12,13]. PIs also cause tremendous financial strain on affected individuals and the US health care system, with an estimated yearly expenditure of US $26.8 billion on hospital-acquired PIs [14].

The burdens and challenges of living with a PI may go unrecognized or unaddressed by the health care teams caring for individuals with PIs [8,11]. Patient-centered care, which incorporates patients’ and caregivers’ perspectives, preferences, needs, and goals, is essential for addressing patients’ challenges and improving treatment adherence and patient outcomes [15,16]. An interventional study of hospitalized patients admitted for acute medical or surgical treatment comparing usual care with patient-centered interventions revealed that a patient-centered educational intervention resulted in greater confidence in medication adherence [17]. Similarly, an interventional study conducted in an inpatient ward specializing in care for patients with neurological, orthopedic, or musculoskeletal conditions revealed that the active involvement of patients and their colleagues during nursing clinical handovers resulted in a decrease in hospital-acquired complications [18]. Thus, understanding the perspectives of individuals living with PIs and their caregivers may provide insights into improving the delivery of patient-centered PI care [15,16].

Over the past few decades, social media has become a vital resource where communities of individuals living with health conditions and their caregivers from around the world share unfiltered opinions, seek health information, and connect with one another [19]. Social media platforms such as YouTube, X (formerly Twitter), and Reddit are popular forums for individuals to seek social support and health information or share their personal experiences with their health conditions and subsequent treatment [20]. The increasing use of social media by patient and caregiver communities presents a valuable opportunity for researchers to elucidate candid patient and caregiver perspectives and experiences as reported on social media sites [19]. This is made possible through social media listening (SML) [21,22], a research method that involves performing a systematic search of public social media platforms. SML uses tools such as Talkwalker, Salesforce Social Studio [23,24], and X Pro (formerly TweetDeck) to identify real-world, firsthand accounts of individuals’ experiences with disease and treatment, quality of life, and unmet needs [23,25].

SML can be used as a complementary research method alongside traditional qualitative or quantitative studies and has been shown to yield several benefits [19]. Given that patient recruitment is not required, SML can reduce recall and reporting biases that may be present in interviews or retrospective data collection methods by removing the researcher from the discussion [19]. SML also allows researchers to collect data from patients without geographic restrictions, providing large-scale insight into a topic [19]. SML has been shown to be an effective method of understanding patient and caregiver perspectives, unmet medical needs, and adherence to treatment that may not have been previously identified in the literature [23-28]. For instance, Perić et al [23] explored the needs and lived experiences of patients with graft-versus-host disease by using Talkwalker to search for relevant posts on Twitter, Facebook, Instagram, and YouTube. Similarly, Kline et al [24] used Salesforce Social Studio and Talkwalker to understand unmet needs, barriers to treatment, patient journey, and treatment options for patients living with amblyopia. Other similar studies have investigated individuals’ experiences with various medical conditions, providing critical insights into individuals’ quality of life, treatment, and perception of their quality of care [27-30].

While previous studies have explored the challenges faced by individuals living with PIs and caregivers who provide support [8,11,13,31], their perceptions of their role in PI care [9,16,32], as well as their knowledge, information needs, and preferences for PI education [15,33-35], none have used an SML approach to explore their experiences. Given the benefits of SML and the pervasive use of social media in the present day, SML may provide important, unique, and unfiltered insights into the experiences, challenges, and needs of individuals with PIs and caregivers globally that may not be elucidated from traditional studies. Thus, our study aimed to capture the experiences, challenges, and needs of individuals with PIs and caregivers using SML, an emerging research method that could reveal aspects of the PI experience not easily observed in controlled research environments.

## Methods

### Study Design

This study implemented a noninterventional qualitative descriptive approach to analyze publicly available social media posts made on X (formerly Twitter), Reddit, and YouTube using SML software Awario and X Pro. The study design was conceived by postgraduate students and scientists with extensive experience in both qualitative and quantitative research. We used the SRQR (Standards for Reporting Qualitative Research) guideline [36] to draft this manuscript and the SRQR reporting checklist [37] when editing (Checklist 1).

### Search Strategy

#### Overview

Between May and June 2023, comprehensive searches were conducted on Reddit and YouTube using Awario. Searches were simultaneously conducted on X using X Pro. Awario and X Pro enable users to search web-based platforms for phrases and keywords in public posts in a similar manner to a literature search [38,39]. Both Awario and X Pro identified English-language posts made between January 1 and December 31, 2022. X, Reddit, and YouTube were selected as popular open-access social media websites where users commonly share personal opinions and experiences, enabling us to feature the perspectives of a wide range of individuals. Predefined Boolean search strings were used to identify relevant posts on each social media platform, and Boolean operators (AND/OR) were used to combine each keyword within the strings (Multimedia Appendix 1). The search string included key terms related to pressure injury and alternate words for pressure injury, such as “bedsore” and “pressure ulcer,” as well as care recipients’ or caregivers’ identities (ie, grandparents, parents, sister).

#### Definitions

A “user” or “social media user” referred to individuals with lived experience (ie, individuals who had experience living with a PI), caregivers (ie, individuals who provided direct, unpaid care to someone with a PI), or observers (ie, individuals who were not explicitly a caregiver but had witnessed someone else’s PI experience as their friend or relative) who posted content on social media. Observers were distinguished from caregivers by their level of participation in providing PI care: caregivers explicitly provided care for someone with a PI, whereas an observer did not provide direct care for an individual with a PI but appeared to be actively involved in the life of an individual with a PI (eg, friend, colleague, or family member). A “post” was defined as any social media content identified through the search. A “mention” was defined as a reference to a specific topic within posts, which was used for coding in the analysis.

#### Inclusion and Exclusion Criteria

Social media posts from X, Reddit, and YouTube were included for analysis if they were written or recorded in English by individuals with lived PI experience, caregivers, or observers. In order to be included in the study, the social media post had to contain content relating to personal experiences with PIs (ie, experiences of living with a PI, caring for someone with a PI, or observations of another person’s experience with PIs). There were no restrictions in terms of the user’s geographic location.

Posts were excluded if they mentioned the development of a PI from medical masks or if the user did not fit the definition of an individual with lived PI experience, caregiver, or observer. Posts were also excluded if the user was a paid caregiver or health care professional (HCP) and if the post contained product endorsements or advertisements.

#### Data Screening

The results of each Boolean search were entered into a Microsoft Excel spreadsheet. Each X, Reddit, and verbatim transcript of the YouTube post was manually reviewed for relevance based on the inclusion and exclusion criteria before being input into a separate spreadsheet for content analysis. Any posts that did not meet the inclusion criteria or the exclusion criteria (eg, product endorsements) were removed. Duplicate posts that contained identical text were identified using the sort function of the spreadsheet and removed manually by researchers. To ensure interplatform data consistency, we applied common inclusion and exclusion criteria across platforms. Screening and verbatim transcription were performed in duplicate by two independent researchers to enhance trustworthiness.

#### Data Collection: Post Characteristics

Users’ relationships with individuals living with a PI, geographic location, and intended audience were documented whenever such information was available from the users’ social media profiles or could be inferred from the posts. Otherwise, users were classified as “unknown.” Post details including the posting date, retrieval date, source platform (ie, X, Reddit, or YouTube), post format (ie, video, forum post, or tweet), and post hyperlinks were also documented on a Microsoft Excel spreadsheet.

### Summative Content Analysis

#### Overview

Segments of text within each social media post were assigned a label (ie, code). Given that multiple segments of text within each social media post may have been present, social media posts were often assigned more than one code. Summative content analysis was used [40,41] through inductive coding; theme generation; and code, subcode, and theme frequency measurement.

#### Theme Generation

Inductive coding was used to formulate a comprehensive codebook. Primary codes, corresponding subcodes, example posts for reference, and code definitions were determined through an iterative process. The initial creation of codes and subcodes was performed independently by four research team members (PM, Pilar Tabuenca [PT], Uchechukwu Akunna [UA], and SG) based on post content, and the final codebook was developed following an exhaustive discussion by the team and the refinement of initial codes and subcodes. Disagreements were resolved through a discussion between coders or by an additional coder, if needed. Codes and subcodes were assigned to the relevant posts on a Microsoft Excel spreadsheet. Emotion codes were assigned to each post based on Plutchik’s emotion wheel [42], which identifies eight primary emotion codes—joy, trust, fear, surprise, sadness, disgust, anger, and anticipation—along with their combinations. To ensure the reliability of the coding, these code assignments were later double-checked by four independent researchers (AI, AAO, IT, and JZS) and input into NVivo 12 (QSR International), a qualitative data management software.

The codes and subcodes were then consolidated into major themes representing the experiences of individuals with lived PI experience as reported on social media following further discussion by the research team. Posts were double coded under the same theme when they reflected multiple aspects of the theme; for instance, posts often described both personal impacts and perceived quality of treatment that were encountered while caring for a PI, which both fell under the theme of “Challenges experienced when living with or caring for a PI.” As a result, the number of mentions (ie, codes) per theme surpassed the number of total posts in some cases.

#### Frequency Measurement

Code, subcode, and theme frequencies were calculated using NVivo 12. Frequency was similarly calculated and summarized for users’ demographic information (ie, geographic location, intended audience, and relationship to the individual living with a PI).

### Ethical Considerations

This study was exempt from human subject research ethics review because all data were obtained from public, internet-based sources, as per University Health Network [43] and University of Toronto [44] policies. No human participants were directly involved in this study, and as such, compensation was not provided. Study data were deidentified, and any identifiable information was removed from the study analysis, manuscript, figures, and tables.

## Results

### Search Strategy and Data Screening

**Figure 1.**
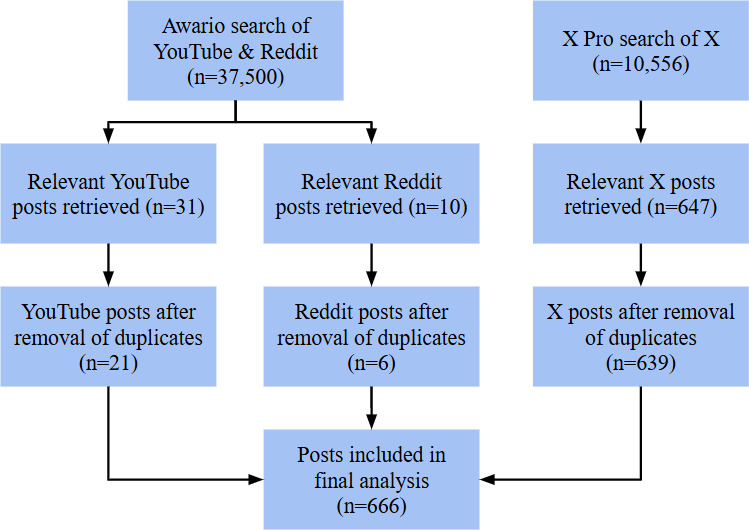
Flow diagram depicting the process of selecting relevant English-language social media posts made between January 1 and December 31, 2022, using social media listening (SML) software programs Awario and X Pro.

The initial search retrieved 37,500 and 10,556 posts from Awario and X Pro, respectively (Figure 1). After three independent researchers (PM, PT, UA) manually screened the posts, 41 relevant posts were identified from Awario, and 647 relevant posts were identified from X Pro. Duplicate posts were then removed. Ultimately, 639 X posts, 6 Reddit posts, and 21 YouTube videos were identified as relevant to the study objectives.

### Post Characteristics

**Figure 2.**
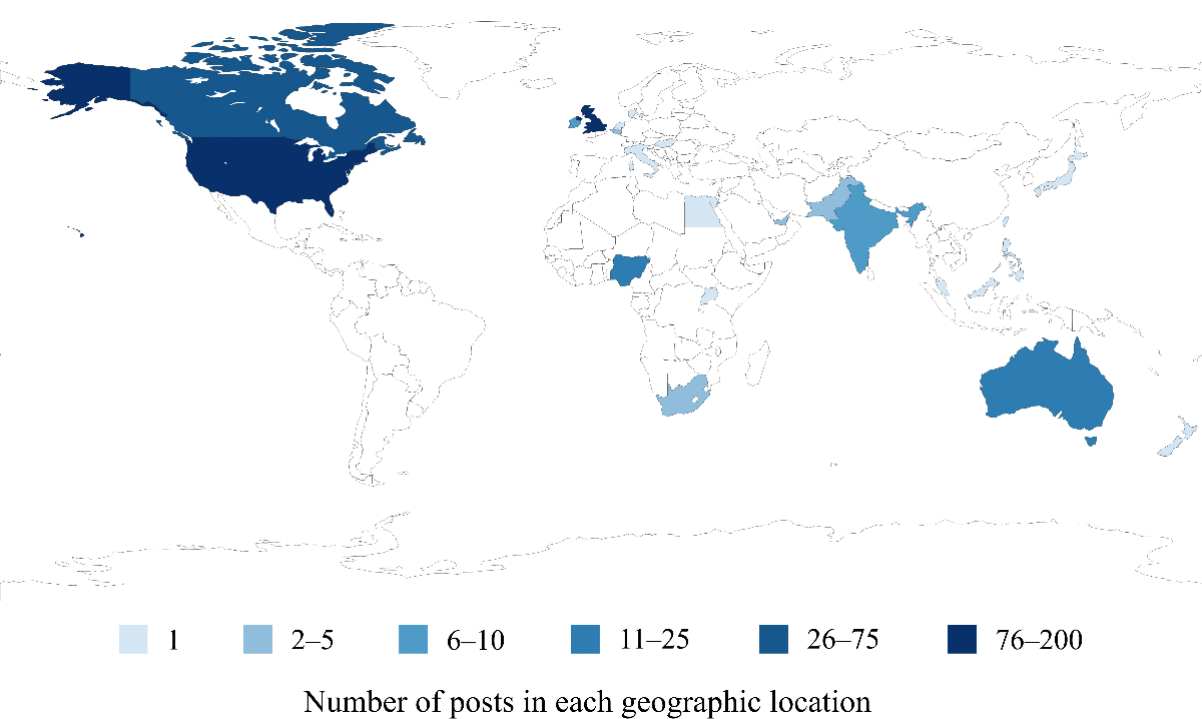
Heat map displaying the distribution of social media posts according to geographic location.

Our study identified 498 unique social media users across X, YouTube, and Reddit who generated a total of 666 posts. Of the 666 posts, 361 (54.2%) were posted by observers, 219 (32.9%) were posted by caregivers, and 86 (12.9%) were posted by individuals with lived PI experience. Caregivers and observers were often the child or other relative (eg, grandchild, sibling, parent) of the person with a PI. Most posts (n=631, 94.7%) were intended for the general public, although a few were targeted at specific health care institutions or other individuals (ie, governments, support groups). Most posts (n=481, 72.2%) were intended to share information about PIs with others (eg, warnings, knowledge about PI progression, causes, and complications), while some posts were intended to blame or advocate for others. Posts most frequently originated from the United States (n=170, 25.5%), the United Kingdom (n=150, 22.5%), or Canada (n=62, 9.3%), as seen in Figure 2.

### Overview: The Pressure Injury Experience

Table 1 displays characteristics related to the PI experience, including the description of the PI, risk factors and causes of PIs, PI symptoms, the setting where the PI occurred, PI management, and commentary about PI healing. In posts (n=666), users frequently discussed the severity of the PI (n=120, 18%) and the location of the PI on the body (n=116, 17.4%). Several users described comorbidities (eg, dementia, obesity) as a risk factor for developing a PI (n=105, 15.8%), and many users cited a lack of care from HCPs or caregivers as the direct cause of their PI (n=156, 23.4%). Additionally, pain and discomfort were commonly reported symptoms (n=56, 8.4%). Many PIs were reported to have been hospital-acquired (n=197, 29.6%), and the most commonly reported PI management strategy was local wound care (n=99, 14.9%; eg, debridement, cleaning). A few users described the healing process of their PI (n=41, 6.2%).

**Table 1.** Frequency of posts that reported elements of the pressure injury (PI) experience, including PI description, risk factors, causes, symptoms, setting, management, and healing^a^.

Topic	Posts, n	Relevant quotes
Pressure injury description		
Severity	120	“… my back, bottom & legs were a mass of pressure sores, two so severe that you could fit a large man's fist into the holes.”
Location on body	116	“My Mum had a bedsore at the bottom of her spine right through to the spine. It killed her …”
Size	50	“… she had heel pressure ulcers the size of a silver dollar and deep ...”
Appearance	36	“… her bottom became an un-stageable bedsore. After surgery to remove [the] blackened and dead flesh, you could actually see her lower spine.”
Number	34	“I’m alive, but I am dealing with three separate pressure ulcers …”
Duration	33	“Almost seven years of dealing with that pressure sore and it's finally healed …”
Risk factors of pressure injury		
Comorbidities	105	“She keeps getting UTIs and now she has a pressure sore that's slow to heal. She needs a hoist to get up. Her dementia is getting worse ...”
Immobility	50	“… She was essentially immobile, and fat. It took three hospital staff every time she needed to be repositioned to prevent bedsores.”
COVID	15	“My mom got COVID… A few months later, she got an infection from a bedsore, got sepsis, and passed away. I believe COVID weakened her immune system …”
Body mass	15	“[He] was about 1200 pounds, bedridden, and ended up having a huge wound as a result of a bedsore.”
Thin skin	7	“My mom went in hospital and got pressure ulcers like that within days ... Her skin was very thin.”
Cause of pressure injury		
Lack of care	156	“She has two pressure sores on one foot as the carers in the home didn’t move her when she was out of it for a couple of days.”
Immobility	103	“... [My grandpa was] completely paralyzed. Ended up dying from a bedsore because of it.”
Comorbidities	63	“…due to my spin a (sic) bifida and lack of sensation in some areas of my body I developed pressure ulcers on my sacrum and was hospitalized for wound care and sepsis.”
Equipment-related	39	“I got a pressure sore on the back of my heel from my cast …”
Symptoms related to pressure injury		
Pain or discomfort	56	“My gran was constantly crying due to pressure ulcers …”
Bleeding or exudate	12	“… We are bringing my dad to see someone about some bedsores we are worried about because they’re starting to bleed.”
Odor	3	“… I could smell the infection as soon as I entered the room.”
Setting where pressure injury occurred		
Hospital (ie, ICU^b^, ER^c^)	197	“... I’ve seen how they treated my mother in the hospitals ... She came home with a bedsore.”
Care home	64	“Just like my loved one in LTC a couple of years back … She was yelling and crying out and the staff dismissed it as a regular dementia behavior … She ended up with a terrible pressure sore …”
Home	63	“Got my first pretty bad pressure sore since coming home, I do not want to go back to the spinal unit man.”
Pressure injury prevention and management strategies		
Local wound care	99	“... [My dad] had surgery to debride bedsores.”
Use of equipment (eg, cushion, mattress)	38	“... [My mom] had a scary bedsore & was given a gel pad for chair & bed. I have a gel mattress which is great for not getting bedsores …”
Offloading	21	“... You are the person who turns her over every 2 hours or she will get bedsores.”
Bed rest	14	“[He] has been trying to heal a pressure sore by being on bed rest for nearly a year.”
Inspection or assessment	14	“… I do skin checks every morning when I get up, every evening when I go to bed, and every time I go to the bathroom, I check the vulnerable areas.”
Medication	14	“Just beginning my 3rd month in hospital as a result of a pressure sore. 9 weeks of IV antibiotics due to osteomyelitis.”
Nutrition	11	“When I was recovering from severe damage related to my pneumonia/sepsis episode (stage 3 pressure ulcers, crushed immune system, etc.) they made me aim for 120 [grams of protein] every day. Three medical Ensures, special feeding tube formula ...”
Healing of pressure injury		
Healing process	41	“At the start the pressure sore needed 3 packing strings. Now, only half of 1 fits in.”
Healing time	25	“My grandma (86) got a really bad bedsore … After nearly 20 months, it’s finally almost healed.”
Lack of healing	22	“I have a pressure sore right now that’s been open for several months and not close to healing …”
Worsening	12	“My downfall was a pressure sore which rapidly went from bad to worse …”

a Some posts contained multiple constructs of the PI experience.

b ICU: intensive care unit.

c ER: emergency room.

### Theme 1: Challenges Experienced When Living With or Caring for a Pressure Injury

Within the 666 posts, there were 807 mentions related to the challenges associated with living with a PI, caring for someone with a PI, or receiving care for a PI. A total of 423 out of 807 (52.4%) mentions identified the personal impact or impacts of living with a PI or caring for someone with a PI, with 221 out of 423 (52.2%) mentions discussing the challenges associated with PI complications, such as infection and death. A total of 158 out of 423 (37.4%) mentions related to the personal impact of living with a PI reported a negative impact of PI or caregiving on overall functioning (eg, work or employment, mobility, daily activities) and perceived quality of life. A total of 23 out of 423 (5.4%) mentions related to the personal impact of living with a PI reported negative financial impacts (ie, struggling to afford treatment), and 21 out of 423 (5%) discussed negative mental health impacts such as depression and social isolation.

*My dad died from overwhelming sepsis due to osteomyelitis caused by his bedsores [and] of course everyone in the ICU had [pneumonia]. I checked his chart each day [and] it was sad to see signs of life slowly slipping away. Pressors couldn’t keep him up.*

*Mum has to stay in bed most of the day cause of pressure sore/open wound, we only transfer her to chair for a few hours.*

Additionally, there were 241 out of 807 (29.9%) mentions of the perceived quality of PI treatment received, with 205 out of 241 (85.1%) mentions describing poor care practices such as lack of care and misdiagnosis from HCPs that commonly led to PI complications for the individual. A total of 36 out of 241 (14.9%) mentions related to the perceived quality of treatment reported witnessing poor attitudes of caregivers and HCPs when treating the individual with a PI.

*I had no idea [my mom] was suffering from a level 4 bedsore [because] the facility didn’t tell me. The hospital told me. Shameful. This is the norm in most LTC facilities. This has to change. Now. #nursinghomeneglect.*

*I haven’t even mentioned some of the worst parts of this. My mom developed multiple new pressure ulcers at [healthcare facility]. I’ve had nurses and therapists privately tell me how bad things are at [healthcare facility]. I was told a nurse practitioner sighed in disappointment that my mom was improving.*

Furthermore, there were 102 out of 807 (12.6%) mentions related to perceived health care system issues when accessing services to treat PIs. Of these 102 mentions, 57 (55.9%) reported issues with accessing equipment or services (eg, difficulties obtaining pressure mattresses, long hospital wait times) and 26 (25.5%) mentions described prolonged hospitalization or inability to be discharged due to these challenges with health care access. Additionally, 19 out of 102 (18.6%) mentions related to perceived health care system issues described witnessing critical staffing shortages at health care facilities that contributed to the deterioration of the individual with lived PI experience.

Please help. *Nobody will give us an air mattress for my elderly sick bed bound father! It’s a disgrace when he is starting to get bedsores. He’s under palliative care but without complex needs!!!!*

*My poor dad ended up with a giant bedsore while on the barely staffed dementia ward at a [location] care home*. *He died of sepsis because of it. I was always stunned that there never seemed to be anyone up there working! So effed up!*

Finally, there were 41 out of 807 (5.1%) mentions related to a perceived lack of knowledge from HCPs, caregivers, or individuals with lived PI experience. Of these 41 mentions, 22 (53.7%) described a perceived lack of HCP’s PI knowledge that contributed to the development or worsening of a PI. Moreover, 19 out of 41 (46.3%) mentions related to a lack of knowledge indicated the individual with lived PI experience or caregivers’ lack of knowledge related to PIs or their prevention and management.

*My mother had a horrendous [suppurating] bedsore on the back of her head after 8 weeks solid lying prone. We didn’t know what it was but asked a nurse who shrugged and said, “Oh, that’s weird. I don’t know what it is” and walked off. It took 18 months to heal once home.*

*I can’t wait to look at my birthday messages, but [right now] I’m going to see my grandma [because] she’s going to the hospital [for] bedsores that we don’t know how to take care of.*

### Theme 2: Needs Related to Pressure Injury Prevention and Management

There were 110 mentions about the users’ needs related to PI prevention and management. Of these 110 mentions, 39 (35.5%) described a need for health care support. These mentions shared the users’ need to receive PI care from HCPs, as well as the difficulty in accessing care.

*[I have] infected pressure ulcers. Can’t get ahold (sic) of family doctor. Virtual care clinics are booked up for multiple days—no way to get myself to a clinic.*

*No, I had been trying to get treatment for pressure sores, but both my family members had covid at the time and couldn’t take me to any appointments due to being covid positive.*

Moreover, of the mentions related to the user’s needs related to PI prevention and management, 38 out of 110 (34.5%) mentions were related to a need for educational support. These mentions described users’ need for advice from HCPs, health care organizations, or the general public related to PI prevention or management.

*My dad is in a care home. He has dementia and now bad grade bedsores. Doctor said they are bad and could turn sepsis and [there is] nothing else they can do. Do I need a second opinion? Please help. Thanks.*

*[My dad’s] bedsores are bad which the Doctor has said could lead to sepsis. Any help or advice, please.*

In addition, there were 26 out of 110 (23.6%) mentions related to the need for social support. These mentions often described a need for spiritual guidance, prayers, or words of encouragement.

*Please pray for me, I’m suffering with a pressure sore that won’t go away.*

*I’ve been dealing with a pressure sore for a [little] bit now. Today I found out it’s infected. It’s very triggering as pressure sores is how I lost my legs. Please send good vibes [or] distractions and stuff. I feel awful.*

Finally, there were 7 out of 110 (6.4%) mentions related to users’ need for financial support. In these mentions, users requested assistance with medical bills related to PI prevention or management.

*[Please] help my family to pay my mom’s hospital bill*. *There’s another 16 [days] left for surgery but yesterday my mom got admitted due to sudden bedsores and she’s in pain right now. We [do not have any] income to buy the medication.*

*Please help me. I’m getting pressure ulcers from having to lay in bed a lot due to feeling unwell from my cancer. I started a [GoFundMe] a while ago and it hasn’t got too much traction. I love you all and hope you’ll take a second to boost this.*

### Theme 3: Emotions Experienced Related to Living With or Caring for a Pressure Injury

There were 478 mentions related to emotions when experiencing a PI, including primary emotions (ie, joy, trust, fear, surprise, sadness, disgust, anger, and anticipation) and a combination of these primary emotions (eg, anger and sadness, fear, and anger). Of the 478 mentions related to emotions experienced, negative emotions were frequently mentioned: 91 (19%) of the emotions included anger, 36 (7.5%) included sadness, and 68 (14.2%) included a combination of the two. Combinations of sadness with other primary emotions (eg, disgust, surprise, anticipation) were also frequently observed in 82 (17.2%) of the mentions. In total, 62 (13%) of the mentions included a combination of anger with other emotions. Combinations of fear with other emotions were also common in 54 (11.3%) of the mentions.

*My mother is in aged care. Now transferring to palliative care after neglect. Massive bedsores despite instructions from doctor on how to position her. $600k plus daily fees for neglect and malnutrition that will ultimately see her out. We are beyond furious*.

*I am a mess, honestly. My sister and I take turns everyday feeding and hydrating her. She has a nasty bedsore, bone exposed … We can’t even get her up to take her for a walk. I am just sad*.

*I’ve been TERRIFIED of getting pressure sores for a decade [and] half now, given my super low ability to move even in the bed I’m stuck in. I’m sitting or lying almost 24/7 … I’m super high risk [and] when it finally happens ... The [doctors] don’t care.*

Positive emotions were less frequently mentioned and included joy in 11 out of 478 (2.3%) mentions, anticipation in 5 out of 478 (1%) mentions, or both in 21 out of 478 (4.4%) mentions. Often, these emotions were expressed when describing appreciation for the kindness of others or anticipation related to the PI recovery process or prognosis.

*My heart bleeds seeing my dad this way, bedsore has taken over but your donations kept him till this time. I can’t thank you all enough but I know my God will reward you for your good deeds amen.*

*Today’s goal: I’m hoping to take him on a wheelchair expedition outside his room*-*-maybe even the hospital--for the first time in 2 months! Hopefully his pressure ulcer and spinal pain will allow it.*

*A good day! Nurse says pressure sore looking good, don’t need to bandage it up with padding anymore. I should be able to pick up drugs at Tesco later [and] I think I can try get driving assessed and car adapted so I can drive with foot drop while it’s healing! STOKED!!! #traumarehab*

## Discussion

### Overview

To our knowledge, this is the first study investigating the experiences and perspectives of individuals with lived PI experience as shared through public social media posts. Using SML, we were able to capture the candid experiences and perspectives of individuals with PIs and caregivers that may not be easily gathered from controlled studies. Based on our findings, three major themes were developed: challenges experienced when living with or caring for a PI, needs related to PI prevention and management, and emotions experienced when living with or caring for a PI.

### Principal Findings

#### Perceived Challenges of Living With or Caring for a PI

The most frequently mentioned perceived challenge related to PIs was the personal impact of living with a PI or caring for someone with a PI; most mentions involved challenges associated with complications of PIs, such as infection and death. Previous work notes that individuals living with PIs have an estimated two times higher risk of mortality compared with individuals living without PIs, often due to the development of severe infections [45]. Individuals with PIs are at high risk for bacteremia, which can cause a mortality of over 50% [46]. The findings from this work may help guide the development of strategies to help address this concern among those with lived PI experience.

Social media users frequently mentioned the negative impacts of PI on the daily, physical, occupational, or general functioning of individuals living with PIs. Previous studies on caregivers and those living with PIs identified similar experiences, with individuals reporting a reduction in mobility, independent movement, and quality of life [47-49]. A recent systematic review found that individuals living with PIs often demonstrated declines in physical function and activity, along with low quality of life scores [50]. These deficits are exacerbated by PI complications, such as pain and odor, which may impede the performance of physical and social activities [50]. In alignment with best practices for PI prevention and management, a holistic interprofessional approach should be used to address not only the PI but also the complications of living with a PI to help optimize outcomes.

Perceived poor quality of treatment related to PI management by HCPs or caregivers was reported by social media users. Posts often mentioned poor attitudes of HCPs and caregivers and neglect of the individual with lived PI experience, leading to increased pain experienced by the individual with lived experience, the development or worsening of a PI, infection, and even death. Other studies have highlighted negative attitudes of HCPs toward PI prevention and/or management as well as HCPs who engage in inadequate PI care practices [51-55]. These deficits in practice are potentially related to reported low levels of knowledge of HCPs related to PI prevention and management [56-60]. This sentiment was shared by social media users in this study who reported a lack of HCP knowledge related to PI management.

According to the knowledge, attitudes, and practices model, knowledge may correlate with attitudes and health practices [61,62]. Thus, the reported lack of knowledge among HCPs may have also contributed to the reported negative attitudes and suboptimal care practices reported by social media users in this study. This is supported by previous studies investigating nurses’ attitudes and perceived barriers toward PI prevention and management, which found that inadequate nurse training and knowledge were major obstacles contributing to their negative attitudes and inadequate practices [7,63,64]. Since low knowledge about PI may lead HCPs to adopt poor attitudes and health care practices, the need to ensure that HCPs receive sufficient training in PI prevention and management should be considered.

In addition to a lack of training and knowledge, HCPs have cited a lack of time and equipment (ie, pressure-relieving devices) as prominent perceived barriers to care delivery [51,65-67]. Other studies have found that staffing shortages and inadequate HCP training were persistent barriers to PI prevention practices [68-71]. These findings align with results from the present study, as social media users commonly identified health care system issues, including staffing shortages and lack of availability of equipment (eg, specialized mattresses). Health care system–related barriers appear to be shared globally, and future initiatives should ensure that equipment for PI prevention and management is accessible in order to optimize health outcomes for individuals living with PIs.

#### PI-Related Needs Among Those With Lived Experience

HCPs play a pivotal role in ensuring that the health care needs and goals of the person living with the PI are met [72,73]. Therefore, it is not surprising that the most frequently mentioned need identified by social media users was health care support related to managing PIs. Although best practice guidelines include self-management where people living with PIs assume responsibility for their PI [74], the frequent sentiment of social media users was the need for health care support, highlighting the limitations of self-management alone. Additionally, users often reported a continued need for HCP support, suggesting the need for ongoing provision of services. One potential contributor to the prevalent need for support may be the inaccessibility of PI resources. Previous studies have demonstrated that individuals seeking specialized medical care experience barriers related to long wait times and high treatment costs, making it exceptionally challenging to obtain relevant services [75,76]. Thus, these barriers may impact individuals’ access to satisfactory PI care, driving their self-reported need for health care support.

Social media platforms provide a resource for individuals seeking guidance on managing their illnesses, allowing them to benefit from the advice and experiences shared by others [77,78]. As such, our study found that one of the most commonly expressed needs of individuals living with PIs was their request for advice and information regarding PIs. Social media can be beneficial as an educational resource because authentic accounts of an individual’s illness can make medical information more relevant to the user’s situation and help them to better understand it [78]. However, given the large volume of information available on the internet, the quality of information can vary widely [79] depending on its source and other post characteristics (eg, length of the post, author credentials). A study evaluating the quality of health information shared on online discussion forum websites, such as Reddit, found that posts shared by medical doctors were “of reasonably good quality” [80]. A recent study by Bang et al [81] that examined the quality of PI health information on YouTube found that longer videos produced by a physician and other health personnel contained more accurate and reliable information about PI prevention and management. In contrast, health information on X may not be reliable: a recent retrospective cohort study revealed that X users were approximately 974 times more likely to encounter inaccurate than accurate information about monkeypox [82]. Similarly, another study found that while authenticated authors on X with large followings were more likely to produce accurate posts about COVID-19, over 25% of the posts were inaccurate, indicating that this information may not be reliable for educational purposes [83]. Future studies should evaluate the quality of PI health information on various social media platforms and elucidate the factors impacting information quality to provide guidance for users regarding where and how to obtain reliable and accurate information.

Social media serves not only for sharing individual experiences but also for offering and receiving support [78]. In our study, social support emerged as a frequently mentioned need, which aligns with previous study findings that many caregivers of adults with cancer expressed a desire for social support in the form of prayers and patient visitation [84]. Individuals living with PIs are particularly vulnerable to social isolation due to the challenges associated with immobility and body image issues due to characteristics of PIs such as exudate and malodor [7,85]. Hence, social media may be a valuable resource for those living with PIs, allowing them to connect with others who can offer support. This notion is further supported by a study on social media use among adults during the COVID-19 pandemic, which highlighted how social media platforms can be major sources of social support amid isolation, ultimately aiding in the reduction of loneliness [86]. Future work should consider developing interventions to address the social isolation that individuals living with PIs may face.

#### Emotions Experienced as a Result of a Pressure Injury

In this study, anger, sadness, and a combination of the two emotions were the most frequently cited among social media users. This aligns with previous research findings that individuals with lived PI experience and caregivers often feel negative emotions such as anger, frustration, and sadness due to the lengthy healing process of PIs, lack of care received, and PI symptoms such as pain [7,32,50,87-89]. In the qualitative study of Latimer et al. [32] that explored the perceptions of individuals with PIs regarding their role in PI prevention, participants reported feeling angry and frustrated about failing to receive appropriate PI preventative care and experiencing negative interactions with HCPs. These findings suggest that the PI experience is often distressing and can incite intense negative emotions in those living with or caring for a PI. However, it is important to note that social media users tend to engage with and share negative emotions and experiences (eg, anger and distress) more frequently than positive ones on these platforms [90-95]. In addition, studies have shown that those in the online health community may be more likely to post negative content to seek emotional and informational support [96,97]. The platform X in particular has been associated with messages displaying highly negative emotions [92]. Given that X was our primary source of data, the potential likelihood of predominantly negative experiences reported on X was high, and as a result, our findings may be skewed. Nonetheless, our findings provide valuable insights into individuals’ experiences living with or caring for a PI, highlighting the negative emotional impact of the pervasive challenges associated with preventing and managing PIs.

#### Implications for Digital Health

Our study identified the candid experiences, challenges, and needs of individuals with PIs and their caregivers, which have several actionable implications for digital health. In this study, individuals living with PIs and their caregivers frequently expressed a need for information and advice regarding PIs and PI care. Additionally, social media users often reported a continued need for HCP support throughout the PI journey, which they lacked access to. These findings suggest that researchers and clinicians should prioritize the development of accessible online PI educational materials or platforms for individuals with PI and caregivers. Online PI resources or platforms (eg, websites, mobile health apps, social media pages) could provide contact information for HCPs or health organizations, enabling individuals with PI and/or their caregivers to access PI information or support when needed. Disseminating critical health information in a centralized and accessible forum may help promote health literacy among individuals with PIs and caregivers who rely on social media sources to supplement knowledge gaps. In addition, given the widespread use of social media today, it may also be useful to develop social media campaigns to educate the general public about PIs to combat general misinformation.

SML allowed us to identify patterns in the experiences and struggles of individuals with PI and their caregivers, such as a perceived lack of adequate PI care received and lack of knowledge among HCPs. These issues may subsequently be targeted and addressed by relevant regulatory or health entities to improve patient experiences and outcomes. As such, we note that SML may be used in the future to complement digital health surveillance technology, as researchers may utilize SML to observe and track ongoing trends in the reported challenges and needs of individuals with PI and caregivers on social media. The information gathered by SML can generate important longitudinal insights into whether prevailing issues are being adequately addressed and what critical areas of health care improvement health care organizations and policymakers should focus on.

### Limitations

While our study is strengthened by a rigorous screening and coding process to analyze and interpret social media data, we acknowledge several limitations. First, since researchers hand-coded all social media posts, it is possible that some codes were missed due to human error affecting the accuracy of the reported frequencies. However, this process was carried out through a meticulous process (ie, researchers held iterative discussions to determine codes and code definitions, with coding performed in duplicate), so this is not a notable concern. Additionally, we broadened search terms in our Boolean search strings to widen our search and maximize the number of relevant posts. However, we acknowledge that some of these search strings could be streamlined and improved upon in the future for optimal retrieval and efficiency in our analysis. Furthermore, only English-language posts were included, which largely limited our search results to content from English-speaking countries. Moreover, most of the posts included were created by users in the United States, meaning our findings may predominantly reflect the health care experiences of individuals living in this country. Another potential limitation is that we only searched YouTube, Reddit, and X; a broader range of social media platforms may have yielded more posts for inclusion. Additionally, we found that X yielded a disproportionately large number of posts compared with YouTube and Reddit, which may have skewed our analysis toward findings from X. Also, the format of posts across the selected platforms was very different and thus difficult to collectively analyze. As such, future research using SML may benefit from selecting more comparable social media platforms to facilitate analysis or analyzing posts from each platform separately. Additionally, we were unable to obtain a complete demographic profile of social media users (eg, age, gender, or sex) due to the limited demographic information available on social media user accounts. Since we analyzed posts from observers, caregivers, and individuals with lived experience as a homogeneous group, differences between the experiences, challenges, or needs of each subgroup were not elucidated. Future studies may benefit from subgroup analysis to identify unique findings for caregivers and individuals with lived experience. Finally, we acknowledge that there may be sampling bias present in our study, as social media users are often younger and more technologically literate [98]. Also, while we did not restrict our sample in terms of geographic location, we acknowledge that there are inequities in technology access and thus ability to use social media platforms [99]. These limitations may reduce the generalizability of our findings.

### Conclusions

Our study used SML to explore the unfiltered experiences shared by individuals affected by PIs (ie, individuals with lived PI experience, caregivers, and observers) across social media platforms X, YouTube, and Reddit. We found that many users described challenges associated with PIs and PI care related to the negative personal impacts of living with a PI and receiving inadequate care from HCPs, highlighting the need to address these issues and identify barriers to appropriate PI care. Additionally, social media was often used to request or express a need for health care support and education, suggesting a critical need for supportive and educational resources for those affected by PIs. Finally, the most frequently observed emotion across posts was anger, consistent with previous findings that individuals with lived PI experience and caregivers often experience negative emotions about the PI experience. Our findings provide valuable insights into the candid experiences of individuals living with PIs, uncovering several gaps in care and research that require intervention to improve the well-being of these individuals. Clinicians, policymakers, and researchers should prioritize addressing the prominent challenges and needs identified from this work to optimize PI care delivery and patient outcomes.

## Supplementary material

10.2196/76682 Multimedia Appendix 1 Keywords, phrases, and Boolean operators used on social media listening software Awario and X Pro to identify relevant Reddit, YouTube, and X posts.

10.2196/76682 Checklist 1 SRQR checklist.

## Abbreviations

HCP: health care professional
PI: pressure injury
SML: social media listening
SRQR: Standards for Reporting Qualitative Research
