# Supplementary material for: Using Social Media Listening to Understand the Pressure Injury Experience: A Qualitative Descriptive Study
Source: JMIR Nurs. 2026 Jun 16;9:e76682. doi: 10.2196/76682 (PMC13271712; doi:10.2196/76682)
Supplement: Multimedia Appendix 1 [file nursing-v9-e76682-s001.docx]

**Multimedia Appendix 2. Keywords, phrases, and Boolean operators used on SML software Awario© and X Pro to identify relevant Reddit, YouTube, and X posts.**

| **Source** | **Search terms** |
| --- | --- |
| **Awario©** | 1. (“Caregiver” OR “caregivers” OR “care giver” OR “care givers”) AND (“pressureinjury” OR “pressure injury” OR “pressure injuries” OR “pressureulcer” OR “pressure ulcer” OR “pressure ulcers” OR “bedsore” OR “bedsores” OR “bed sore” OR “bed sores” OR “experience” OR “experiences”) 2. (“pressure injury” OR “pressure injuries” OR “pressure ulcer” OR “pressure ulcers” OR “bedsore” OR “bedsores” OR “bed sore” OR “bed sores”) AND (“experiences” OR “experience” OR “family member” OR “family members” OR “parent” OR “parents” OR “mom” OR “mum” OR “mother” OR “dad” OR “father” OR “sibling” OR “siblings” OR “brother” OR “sister” OR “grandparents” OR “grandma” OR “grandpa” OR “caring for” OR “taking care” OR “looking after” OR “helping” OR “watching” OR “care recipient” OR “care recipients” OR “Caregiver” OR “caregivers” OR “care giver” OR “care givers”) 3. (“experience” OR “experiences”) AND (“pressure injury” OR “pressure injuries” OR “pressure ulcer” OR “pressure ulcers” OR “bedsore” OR “bedsores” OR “bed sore” OR “bed sores” OR “care recipient” OR “care recipients”) 4. (“care recipient” OR “care recipients”) AND (“pressure injury” OR “pressure injuries” OR “pressure ulcer” OR “pressure ulcers” OR “bedsore” OR “bedsores” OR “bed sore” OR “bed sores” OR “experience” OR “experiences” OR “Caregiver” OR “caregivers” OR “care giver”) |
| **X Pro** | 1. (“pressure injury” OR “pressure injuries” OR “pressureinjury” OR “pressureinjuries”) 2. (“pressure ulcer” OR “pressure ulcers” OR “pressureulcer” OR “pressureulcers”) 3. (“pressure injury” OR “pressure injuries” OR “pressureinjury” OR “pressureinjuries”) AND (“caregivers” OR “caregiver” OR “care giver” OR “care givers”) 4. (“pressure ulcer” OR “pressure ulcers” OR “pressureulcer” OR “pressureulcers”) AND (“carerecipients” OR “carerecipient” OR “care recipient” OR “care recipients”) 5. (“pressure injury” OR “pressure injuries” OR “pressure ulcer” OR “pressure ulcers” OR “bedsore” OR “bedsores” OR “bed sore” OR “bed sores”) AND (“experiences” OR “experience” OR “family member” OR “family members” OR “parent” OR “parents” OR “mom” OR “mum” OR “mother” OR “dad” OR “father” OR “sibling” OR “siblings” OR “brother” OR “sister” OR “grandparents” OR “grandma” OR “grandpa” OR “caring for” OR “taking care” OR “looking after” OR “helping” OR “watching”) 6. (“pressure sore” OR “pressure sores” OR “pressuresore” OR “pressuresores”) |
